# Using the Unmanned Aerial Vehicle Delivery Decision Tool to Consider Transporting Medical Supplies via Drone

**DOI:** 10.9745/GHSP-D-19-00119

**Published:** 2019-12-23

**Authors:** Margaret Eichleay, Emily Evens, Kayla Stankevitz, Caleb Parker

**Affiliations:** a FHI 360, Durham, NC, USA.

## Abstract

We developed an unmanned aerial vehicle (UAV) Delivery Decision Tool to help health system decision makers identify their transport challenges and explore the potential utility and impact of UAVs on the broader health system.

## INTRODUCTION

Unmanned aerial vehicles (UAVs) or drones are increasingly explored as a solution to transport challenges for medical goods, including emergency blood supplies, vaccines, medicines, diagnostic samples, and even organs, particularly for “last mile” delivery.^1^^,^^2^ Proof-of-concept tests have demonstrated the technological viability of UAVs to safely transport medical supplies^3^^–^^6^ and keep them within the required parameters for clinical viability.^7^^–^^9^ A few research studies have determined the cost-effectiveness of adding UAVs to specific medical supply chains^1^^,^^10^ or the optimal placement for UAV stations.^11^^–^^13^ Yet very few cases of successful scale-up of UAVs for medical transport exist. Recent work has focused on proving a UAV can successfully complete a flight but has neglected to address health-system integration and long-term sustainability. As a result, decision makers lack information on how to explore the potential utility of UAVs in addressing medical transport challenges and, if determined useful, how to add UAVs to health systems or achieve use at scale.

Successful implementation of any health system change relies on a complex set of factors.^14^ In the case of UAVs, not only will the selected technology need to be appropriate for the distance, weight, and size requirements of the transported items, but (1) it will need to operate within regulations, (2) the concept must be embraced by stakeholders, (3) financial resources need to be available, (4) human resources must be in place, and (5) operational procedures must be developed to effectively work within existing structures. When these aspects are not considered, operations can be delayed, inefficient, or fail entirely. In short, as with any intervention, the impact of UAVs on the broader health system should be considered before implementation.^15^

Using the framework of the World Health Organization health system building blocks,^16^ this commentary describes challenges for integrating UAVs into complex health systems and presents a tool for considering whether UAVs could help address medical transport challenges and how they can be integrated into health systems. Under the assumption that readers may be unfamiliar with UAVs, we begin with some general information (Box).

BOX What Is an Unmanned Aerial Vehicle (UAV)?^1^^,^^4^

- UAVs are aerial vehicles guided without an onboard crew.
- They can be piloted remotely or programmed to fly autonomously.
- A variety of models exist, each suited to different applications based on distance, payload, maneuverability, fuel sources, durability, need for landing, and other factors.

## UAVS FOR MEDICAL TRANSPORT

Routine delivery of medical supplies via UAV is still relatively new. Until recently, there has been only 1 example of routine medical supply using UAV delivery. Since 2016, Zipline has operated drones for the Government of Rwanda, delivering up to 3 liters of blood within 30 minutes to health facilities that request it on demand.^17^ In 2019, however, several other companies received approval to conduct routine flights. Matternet, a company that has conducted hundreds of test flights in Switzerland, routinely transports laboratory specimens within a North Carolina health system.^18^ Alphabet’s Wing will soon be delivering over-the-counter medications, via UAV in Canberra, Australia, after a year and a half of test deliveries.^19^ Yet to date, none of these projects have published on the decision to implement UAV delivery, whether/how they integrated UAVs into existing health systems, or the impact the change has had on health care operations.

In addition to these larger-scale operations, many other pilot projects delivering medical goods are being implemented worldwide,^4^^,^^20^^,^^21^ transporting items such as childhood vaccinations,^22^^,^^23^ automatic external defibrillators,^11^^–^^13^ snakebite antivenom,^24^ tuberculosis sputum samples,^8^^,^^25^ and sterile mosquitoes.^26^ Although these projects are increasingly sharing information about operations, only a few resources for implementing UAV delivery projects exist ^20^^,^^27^^,^^28^ and none explore the feasibility and impact of adding this technology to the broader health system.

Tools to explore feasibility and impact of UAVs in health systems do not yet exist.

## CONSIDERATIONS FOR INTRODUCING UAV TRANSPORT OF MEDICAL GOODS

### Service Delivery

Adding any new product or process to a large existing health system can be challenging. Companies that have been transporting medical goods via drone for multiple years or are preparing to do so have yet to share information about how they integrate their processes with the health system, what processes have to change, how those impact workflows, or the impact they have on health outcomes. Understanding the potential risks and benefits of a systemic change is important during the consideration and implementation of a new technology for evaluating sustainability and the true cost of implementation. Whether the UAV system should be set up in parallel to or integrated into existing structures and systems is also under debate. As with many health innovations, there is concern that integration into existing structures takes too much time; yet, when parallel systems are devised, it fractures the health system, causing informational and operational silos that may result in future inefficiency.

Understanding potential risks and benefits is important when considering and implementing a new technology.

### Health Workforce

Having an adequate number of appropriately trained staff is a constant challenge in many low- and middle-income countries. Depending on the business model used to operate UAVs, adding them to the health system could have impacts on the health workforce. Health professionals may have a role in loading or unloading a UAV; confirming schedules; securing a loading, landing, or dropping site; documenting deliveries; launching the UAV; or instructing the UAV about its next location. Each of these actions would require training and time, so the volume of deliveries is important to consider as well. The tradeoffs between having health professionals perform these functions or hiring others to do it should be evaluated by decision makers before deciding on an operating model. This decision process is not different than typical health workforce considerations, but because the level of training and the steps required differ by UAV system, it is important to consider this information when designing or choosing any UAV delivery system.

### Financing

The tradeoff between flight distance, the amount of weight a UAV can carry, and cost is another barrier to some applications of UAVs in health care. Applications for delivering medical goods in low- or middle-income countries via UAVs have been limited to very lightweight items (less than 5 kg) and relatively short distances (less than 50 km). However, the technology is constantly improving, such that new UAV models might carry 10 kg in weight up to 300 km in distance. The cost of these technologies is positively associated with distance and payload, and the value of UAV delivery to health provision has yet to be established.

Given the tradeoff between weight, distance, and cost, UAV transport will likely supplement medical supply chains, rather than replace road transport.^1^ Understanding the conditions under which UAVs are cost-effective is a critical but complex area for investigation. Logistics management systems traditionally account for the costs of device operation and maintenance, as well as transport time, road condition, warehousing, and staff.^29^ The cost of adding UAVs to the supply chain will be determined by these measures but unique considerations related to UAVs exist.

First, the cost of the devices and the cost of operating and maintaining them vary substantially. Cost models will need to account for the specific UAV system used as well as staff training to operate and maintain them. Secondly, although road condition will not be a big factor in UAV operating costs, weather condition could be. Knowing the impact of wind, humidity, elevation, precipitation, and temperature on supply chain operations will be critical. In addition to direct costs of the technology and service provision, there are opportunity costs. Calculating the value of faster turnaround times for laboratory test results or the value of having a health provider remain in a facility for a day rather than transporting medical goods is difficult to measure but important for considering supplementing systems operations with UAVs. Although a cost-effectiveness modeling tool for UAV supply chains was created in 2019,^27^ it does not yet incorporate the staff and opportunity costs. Full cost accounting methods are still needed.

**Figure:**
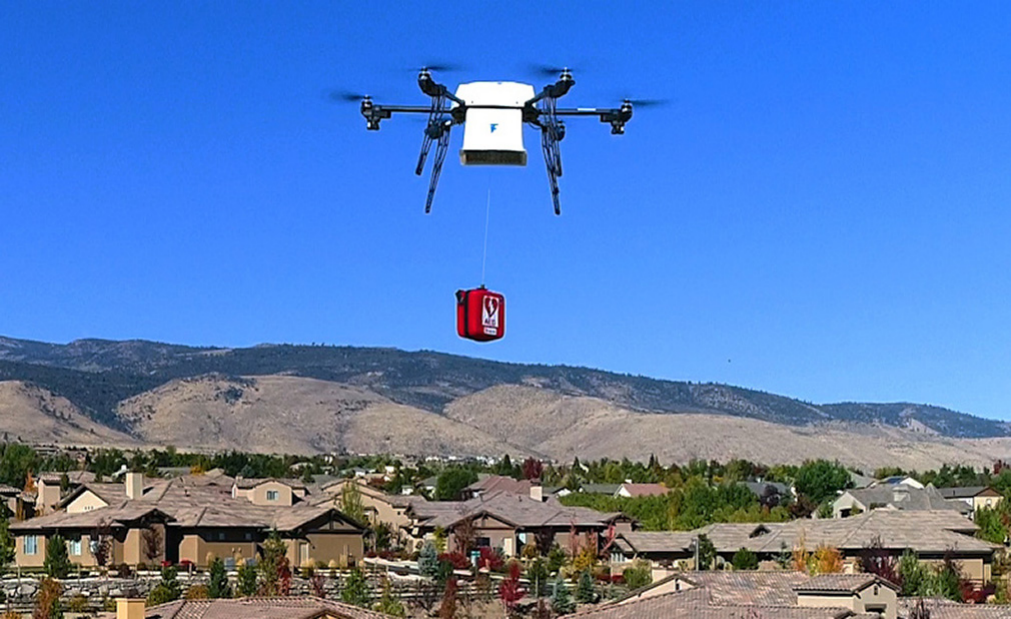
A quadcopter delivers an automated external defibrillator (AED). © 2017/Mollyrose89

In addition to direct costs of the technology and service provision, opportunity costs should be considered.

### Information Systems

Logistics information systems will be substantially impacted by the introduction of UAVs. If UAVs are added to an existing supply chain, all current documentation of what is stocked, transported, and received at multiple locations will have to change to account for method of transport (UAV or other). Because flights might originate from different locations than road route warehouses, these tracking systems will have to be aligned and combined to account for this complexity. Furthermore, logistics systems that optimize routing will require a new set of variables to determine the most efficient routing.

Other health information systems, such as patient and laboratory sample tracking systems, may also be impacted. Ideally, these would all be linked so that a physician who needs to send a patient’s biologic sample to a testing laboratory could easily indicate that in the patient record, which would automatically trigger a request for UAV transport to the appropriate location, and the laboratory would know when it would be received. Although this level of systems integration remains unrealistic in many contexts, starting conversations about potential UAV integration and interoperability of systems early is key to efficient systems design.

Monitoring and evaluation systems are also needed. Supply chains are evaluated based on their ability to provide the correct quantity of goods, maintained within appropriate environmental conditions, to the correct location, on time, and at a competitive cost.^29^ Although the indicators of success are unlikely to change with the introduction of UAVs, measuring how performance changes around the introduction of this new mode of transport will be key to understanding its value. A standardized approach to generate evidence around the introduction of UAVs into public health supply chains is being developed by the Interagency Supply Chain Group's Unmanned Aircraft Systems Coordinating Body and VillageReach.

### Access to Essential Medicines

In the development sector, the goal of most UAV delivery programs will be improving access to health care, including essential medicines. Because access is such an important aspect of health systems operations, it is critical for UAV projects to measure the impact they have on access—not only by assessing how many more people are served, but also by ideally determining whether they are reaching those most in need, thereby measuring equity of access. Although difficult to measure until operations have been conducted for some time, planning for these evaluations early will produce higher-quality information.

### Leadership and Governance

One priority governance issue for UAV use in general, not specific to delivery, is developing national and international regulations in the context of rapidly evolving technology. Air space is highly regulated by civil and international aviation authorities. Anticipating that commercial and federal use of UAVs will add aircraft to the airspace, regulators need to ensure that both large manned aircraft and smaller unmanned aircraft communicate with each other as well as authorities to ensure safe operations. Although UAVs fly at much lower altitudes than most manned aircraft, all aircraft pass through low-altitude space, thereby requiring coordination. The United Nations International Civil Aviation Organization, as well as many national civil aviation organizations, are tackling regulatory issues (e.g., how unmanned air traffic management systems will integrate with manned ones); developing methods to remotely identify UAVs and their operators; standardizing UAV regulations for the humanitarian and development sector; determining who is allowed to remove the threat of an unknown UAV; determining criteria for no-fly zones; and deciding under what conditions UAVs can fly beyond the operator’s line of sight, over people, or at night. Although these issues apply to all UAV flights, they are particularly important to consider before health systems can utilize UAVs for transport, as most applications require long-range transport, either start or end in urban locations, and in emergency situations may not be able to wait for dawn to take flight.

The complexity of health supply chains in low-resource settings requires dedicated stakeholder engagement. Often supply chains involve public, private, and nonprofit actors, national and international entities, siloed transport and tracking systems, and insufficient resources to strengthen them. However, UAV operations will be much more cost-effective if the technology can be used across silos and by multiple institutions.^1^ Although using UAVs for public benefit is often met with enthusiastic response, stakeholders have demonstrated concerns about value for cost, privacy, security, how UAV regulations can be enforced,^30^^–^^32^ and noise.^33^ These concerns can pose real barriers to implementation. Recently, the Government of Ghana’s plan to use UAV services to deliver blood products was opposed by the Ghana Medical Association, a stakeholder that was not sufficiently engaged before decision making and felt the investment misplaced.^34^^,^^35^

Using UAVs for public benefit is often met with enthusiastic response, yet stakeholders have demonstrated concerns, including value for cost, privacy, and security.

Many organizations have been working to ensure that decisions to use UAVs are made with as much information as possible. Over 5 years ago, a collaborative forum was developed to foster discussion and dissemination of UAV delivery work. The Unmanned Aerial Vehicles for Payload Delivery Working Group (UPDWG) currently has over 200 members from public, private, and nonprofit institutions. Recently, a more formal organizing body was created to strengthen coordination between stakeholders and share resources: Interagency Supply Chain Group Unmanned Aircraft Systems Coordinating Body.

As UAV technology has evolved, challenges have arisen in aligning technology partners with stakeholder needs. Ensuring applications of this technology address relevant problems by teams who understand both local contexts and the parameters of the technology remains a challenge. In lower- and middle-income countries, the use of UAVs involves multiple entities: companies who develop and implement the technology, international health and development organizations who implement programs, and national entities who manage the delivery of health care and commodities and implement regulations. We observed a need for a decision-support tool for implementers who are considering UAV technology for transporting medical goods that educates both operators and implementors on relevant considerations.

Ensuring applications of this technology address relevant problems by teams who understand both local contexts and the parameters of the technology remains a challenge.

## TOOL DEVELOPMENT PROCESS

The UAV Delivery Decision Tool was developed using 3 sources of information: a literature review, in-depth qualitative interviews, and expert review of the tool. A variety of readiness assessment tools exist in the literature, yet none of the existing tools offers a practical and directly applicable way to assess the introduction of UAV medical supply transport. Tools that assess organizational readiness for change abound and typically focus on motivation for change or capacity to change at a single institution,^14^^,^^36^ whereas UAV medical transport will require collaboration between multiple institutions (e.g., health facilities, warehouses, regulatory bodies) in both the public and private sectors. Tools related to technology acceptance investigate why and when people use a new technology^37^^,^^38^ and the potential to alter roles and responsibilities within existing structures.^39^ But these tools typically assess existing, not novel, technologies. Drawing from the above review of considerations presented by the World Health Organization health system building blocks^16^ and a review of organizational readiness tools,^14^ we created a list of concepts applicable to the integration of UAVs into the health system and used it to inform the UAV Delivery Decision Tool.

Qualitative interviews were conducted in Nairobi and Turkana County, Kenya, in September 2018 with national- and county-level Ministry of Health officials, health facility staff, laboratory staff, medical supply transport management agencies, and a UAV operator in Kenya. All health personnel interviewed were part of the Afya Nyota ya Bonde project, a 5-year, U.S. Agency for International Development-funded project aiming to improve HIV care and treatment coverage, including commodity management and laboratory services. The project was interested in exploring the cost-effectiveness of using UAVs for transporting laboratory samples in Turkana County, Kenya, where current transport of dried blood spots for HIV viral load testing requires multi-stop transport over 350 km characterized by underdeveloped road networks, insecurity and violence, unreliable transportation, and flash flooding.^40^^–^^43^ Participants expressed interest in the technology and its potential to save time, but they were also hesitant and wanted to understand more about costs and staffing. They mentioned the importance of sensitization, particularly given some of the political and financial sensitivities involved in changing supply chains. Data were also collected on the current costs and frequency of ground-based transport (presented in a separate paper). Information from the literature review and these interviews were synthesized to produce a decision-support tool.

**Figure:**
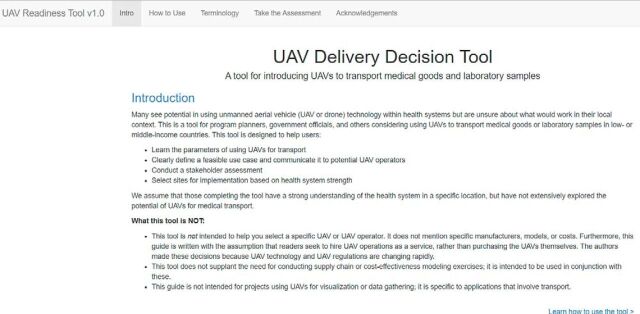
Screenshot of UAV Delivery Decision Tool. © 2019/FHI 360

We requested feedback on the first draft of the tool from members of UPDWG, program directors, and government officials. Most of the feedback received was from UPDWG and was incorporated into the present version.

## TOOL DESCRIPTION

The UAV Delivery Decision Tool (https://fhi360.shinyapps.io/UAVDeliveryDecisionTool/) is a 4-part tool developed in Shiny, version 1.1.0,^44^ an open-access application development package. In Part 1, users define the transport problem they are trying to solve using UAVs and its root cause to determine whether UAVs are a potential solution. Part 2 includes questions regarding transport parameters (origin, destination, distance), the transported goods (weight, dimensions, temperature), and geographic context (terrain, security). Responses to Parts 1 and 2 produce an editable, user-tailored document offering guidance and a clearly defined use case. Parts 3 and 4 contain instructions for completing offline worksheets to identify and analyze stakeholders and to select preliminary sites for conducting UAV operations. Various scenarios can be tested by completing the tool repeatedly and adjusting inputs. We encourage those using the tool to share what they learn from it and from any UAV transport operations through UPDWG (updwg.org) to further encourage collaboration and learning.

## THE WAY FORWARD

Interest in using UAVs to transport medical goods is currently high, but the health sector lacks structured guidance to systematically consider the feasibility, utility, and impact of using UAV transport and evidence regarding implementation. The UAV Delivery Decision Tool is designed to help implementers consider their options and UAV developers to understand the context within which their products need to operate. If a decision to implement UAV delivery is made, gathering evidence on those activities will enable sustainable and careful integration of this new technology that is likely to revolutionize the transport sector in the next decade.

## Notes

**Funding:** This work was supported by FHI 360’s Research and Evaluation Strategic Initiative.

**Peer Reviewed**

**First Published Online:** December 4, 2019
